# Magnetic Ion-Imprinted Materials for Selective Adsorption of Cr(VI): Adsorption Behavior and Mechanism Study

**DOI:** 10.3390/molecules29091952

**Published:** 2024-04-24

**Authors:** Shunfei Li, Siqing Ye, Weiye Zhang, Hongxing He, Yi Zhang, Mingyang Xiong, Yuhan Chen, Mingqiu Wang, Zhifeng Nie

**Affiliations:** Yunnan Key Laboratory of Metal-Organic Molecular Materials and Device, School of Chemistry and Chemical Engineering, Kunming University, Kunming 650214, China; lsf15284548462@163.com (S.L.);

**Keywords:** ion-imprinted polymer, Cr(VI) capture, magnetic separation, density functional theory (DFT)

## Abstract

With the increase of hexavalent Cr(VI) wastewater discharged from industrial production, it seriously pollutes water bodies and poses a risk to human health. Adsorption is used as an effective means to treat Cr(VI), but its effectiveness is affected by pH, and the adsorption performance decreases when acidity is strong. Furthermore, research on the mechanism of Cr(VI) adsorption using DFT calculations needs to be developed. This study focuses on the development of magnetically responsive core–shell nano-ion imprinted materials (Fe_3_O_4_@GO@IIP) through magnetic separation and surface imprinting techniques. Characterization techniques including FT-IR, XRD, and EDS confirmed the core–shell nanostructure of Fe_3_O_4_@GO@IIP. Batch adsorption experiments and model simulations demonstrated the exceptional adsorption capacity of Fe_3_O_4_@GO@IIP for Cr(VI) in strongly acidic solutions (pH = 1), reaching a maximum of 89.18 mg/g. The adsorption mechanism was elucidated through XPS and DFT calculations, revealing that Fe_3_O_4_@GO@IIP operates through electrostatic interactions and chemical adsorption, with charge transfer dynamics quantified during the process. This research provides new insights for addressing Cr(VI) treatment in highly acidic environments.

## 1. Introduction

The heavy metal ions present in wastewater pose a significant threat to the environment. Cr(VI)’s persistence in soil due to its low degradation rate poses a long-term threat to both ecosystems and human health, as it travels through the food chain [1]. Aquatic organisms, including fish and crustaceans, are particularly vulnerable to Cr(VI)’s detrimental effects on reproduction, growth, and behavioral patterns, potentially leading to population downturns [2]. Recent research has elucidated that Cr(VI) can translocate across cell membranes, disrupting redox reactions and causing oxidative stress within cells. This cellular stress subsequently impacts gene expression and functional integrity [3]. The World Health Organization (WHO) sets the maximum allowable limit for Cr(VI) in drinking water at 0.1 ppm [4]. Consequently, the treatment of water contaminated with low concentrations of Cr(VI) is gaining increasing attention.

Currently, the commonly used treatment methods include precipitation, activated carbon adsorption, ion exchange, chemical reduction, electrochemical reduction, and microbial degradation. However, these methods have limitations such as making it easy to produce secondary pollution, having a high cost, high pH value, high energy consumption, and a high environmental impact [5,6,7,8]. In contrast, adsorption is widely regarded as the most promising method for Cr(VI) removal due to its high efficiency, simplicity in operation, economic viability, ease of regeneration, and technical feasibility [9,10]. Hence, there is a pressing need to develop an adsorbent material that is efficient, cost-effective, demonstrates exceptional selectivity, and is adaptable to intricate systems.

Ion imprinting technology, a significant branch of molecular imprinting technology, employs metal ions as templates and involves polymerization through the interaction between functional monomers and metal ions [11,12]. The template metal ions are subsequently removed using an eluent, creating numerous cavities within the polymer that mirror the size, shape, and spatial structure of the template ions. These cavities enable the recognition and selective adsorption of target metal ions. However, conventional ion-imprinted materials face challenges in their recycling and reusability due to their small size [13]. Addressing this issue requires the design and preparation of ion-imprinted materials that facilitate recycling. Magnetic ion-imprinted materials, an advancement in ion-imprinted materials incorporating magnetic particles, exhibit magnetic separation properties, reducing separation and enrichment costs while enhancing stability [14]. Hassanpour et al. synthesized a magnetic Cr(VI) ion-imprinted material for the selective adsorption of Cr(VI) from aqueous solutions with a maximum adsorption capacity of 44.86 mg/g [15]. Nonetheless, the hydrophobic nature of conventional magnetic nanoparticles poses a limitation. Enhancing the hydrophilicity of these materials is crucial for optimizing the performance of magnetic ion-imprinted materials [16]. Graphene oxide, an oxidized form of graphene, has better dispersion in aqueous systems due to its large number of oxygen-containing groups on the surface. This property increases the specific surface area of the material and enhances its adsorption capacity [17]. Neolaka et al. synthesized the IIP@GO-Fe_3_O_4_ material using ion-imprinting and magnetic separation techniques and used it for the removal of Cr(VI) from an aqueous solution, and the adsorption corresponded to a pseudo-secondary kinetic mode with a maximum adsorption capacity of 8.502 mg/g [18]. Therefore, the development of ion-imprinted materials with hydrophilic magnetic properties is essential to advance this field.

This study involved the preparation of magnetically responsive core–shell nano-ion imprinted materials using a combination of magnetic separation and surface imprinting techniques [19]. The structures of the materials were analyzed through various characterizations including FT-IR, XRD, HRTEM, EDS, VSM, and TGA. Batch adsorption experiments and model simulations were conducted to investigate the adsorption capacity, properties, and behaviors of Fe_3_O_4_@GO@IIP and Fe_3_O_4_@GO@NIP. The adsorption mechanism was elucidated through XPS and DFT calculations. Subsequently, the synthesized Fe_3_O_4_@GO@IIP was applied to treat real industrial tail water. The systematic characterization, adsorption mechanism investigation, and application evaluation of Fe_3_O_4_@GO@IIP in this study offer new insights into the practical treatment of industrial wastewater.

## 2. Results and Discussion

### 2.1. Characterization

#### 2.1.1. FT-IR Spectroscopy

**Figure 1:**
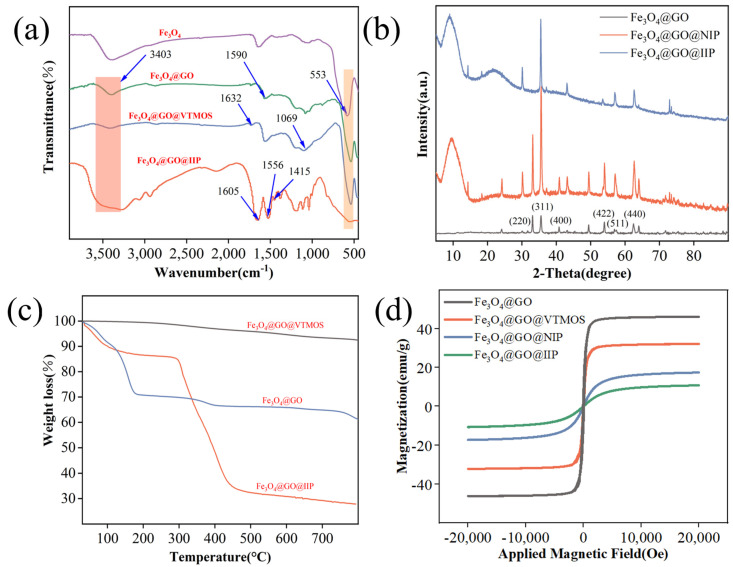
(**a**) FT−IR spectra of Fe_3_O_4_, Fe_3_O_4_@GO, Fe_3_O_4_@GO@VTMOS, and Fe_3_O_4_@GO@IIP. (**b**) The XRD pattern of Fe_3_O_4_@GO, Fe_3_O_4_@GO@IIP and Fe_3_O_4_@GO@NIP. (**c**) The TGA curves for Fe_3_O_4_@GO, Fe_3_O_4_@GO@VTMOS, and Fe_3_O_4_@GO@IIP. (**d**) The VSM curves for Fe_3_O_4_@GO, Fe_3_O_4_@GO@VTMOS, Fe_3_O_4_@GO@NIP, and Fe_3_O_4_@GO@IIP.

The FT-IR spectra in Figure 1a illustrate the changes in the surface functional groups at different stages of the adsorbent synthesis process. Specifically, the peak at 553 cm^−1^ is associated with the Fe-O stretching vibration in Fe_3_O_4_. The peak at 1590 cm^−1^ corresponds to the C=C stretching vibration in the graphene sheets of graphene oxide. This peak is typically associated with the sp2 hybridized carbon atoms in the graphene structure. The peak at 3403 cm^−1^ corresponds to the O-H stretching vibration of the hydroxyl groups present on the graphene oxide sheets. This peak is indicative of the presence of hydroxyl functional groups, which are common in graphene oxide due to the oxidation process used to prepare it. The persistent Fe-O characteristic peaks indicate the effective complexation of Fe_3_O_4_@GO. Furthermore, in the absorption spectrum of Fe_3_O_4_@GO@VTMOS, the peaks at 1069 cm^−1^ and 1632 cm^−1^ represent the stretching vibration peaks of Si-O and C=C in VTMOS, respectively. The peaks at 553 cm^−1^ and 1590 cm^−1^ confirm the successful synthesis of Fe_3_O_4_@GO@VTMOS. Additionally, the absorption spectrum of Fe_3_O_4_@GO@IIP displays peaks at 1415 cm^−1^, 1556 cm^−1^, and 1605 cm^−1^, which are attributed to the broadening vibration peaks of C=N and C=C in the introduced pyridine ring. The peaks at 553 cm^−1^ and 3403 cm^−1^ are related to the Fe-O and O-H broadening vibration peaks in the Fe_3_O_4_@GO substrate material, with a decrease in absorption intensity. This observation validates the successful coating of the Fe_3_O_4_@GO substrate with 4VP [20].

#### 2.1.2. XRD

The structural properties of the Fe_3_O_4_@GO, Fe_3_O_4_@GO@IIP, and Fe_3_O_4_@GO@NIP samples were analyzed using X-ray diffraction (XRD). The XRD patterns, depicted in Figure 1b, were obtained within the 5°–90° range. The diffraction peaks of Fe_3_O_4_@GO were observed at angles of 30.0°, 35.6°, 42.9°, 53.2°, 56.7°, and 62.3°, corresponding to the (220), (311), (400), (422), (511), and (440) crystal faces of Fe_3_O_4_ (JCPDS No. 99-0073), respectively. In contrast, both Fe_3_O_4_@GO@IIP and Fe_3_O_4_@GO@NIP exhibited diffraction peaks maintaining the crystalline facets of Fe_3_O_4_, with additional angular diffraction peaks at 10° attributed to the presence of carbon in GO and tetravinylpyridine. This is in agreement with the FTIR characterization [21,22].

#### 2.1.3. Thermal Stability Analysis

The thermal stability of the samples was assessed through thermogravimetric analysis under N_2_ protection, and the results are presented in Figure 1c. Below 140 °C, all samples exhibited varying degrees of weight loss, primarily attributed to the evaporation of free water within the samples. Specifically, for Fe_3_O_4_@GO, a 30% mass loss at 180 °C was observed, attributed to the elimination of GO at elevated temperatures. In contrast, Fe_3_O_4_@GO@VTMOS displayed a significantly lower mass loss of only 8%, indicating the formation of a SiO_2_ alkylated protective layer through the addition of VTMOS, thereby enhancing its thermal stability. For Fe_3_O_4_@GO@IIP, the mass loss was 15% at 310 °C due to the loss of free and bound water and the decomposition of small amounts of organic matter, followed by a rapid increase after 310 °C due to the decomposition of the polymer on the surface of the adsorbent material. As temperatures exceeded 450 °C, the rate of mass loss decreased as the matrix material decomposed, with a total mass loss of 70% at 800 °C. The thermal stability of Fe_3_O_4_@GO@IIP was specifically evaluated at 310 °C, with it demonstrating good thermal stability up to this temperature. Overall, the thermal stability analysis confirmed the favorable thermal stability of Fe_3_O_4_@GO@IIP [23].

#### 2.1.4. Magnetic Properties

The magnetic properties of the samples were evaluated using a vibrating sample magnetometer (VSM), and the results are illustrated in Figure 1d. The saturation magnetization strengths of Fe_3_O_4_@GO, Fe_3_O_4_@GO@VTMOS, Fe_3_O_4_@GO@NIP, and Fe_3_O_4_@GO@IIP were measured at 46.1, 31.9, 16.8, and 10.6 emu/g, respectively. It is evident that the saturation magnetization strengths exhibit a gradual decrease, attributed to the continuous surface modifications leading to the progressive encapsulation and shielding of the Fe_3_O_4_ nuclei. Furthermore, the saturation magnetization intensity of Fe_3_O_4_@GO@IIP slightly lags behind that of Fe_3_O_4_@GO@NIP, possibly due to the formation of a loose and porous imprinted layer, which offers enhanced shielding owing to its greater thickness. These findings underscore the favorable magnetic response properties of the experimentally synthesized adsorbent, rendering it suitable for magnetic separation applications [24].

#### 2.1.5. HRTEM

**Figure 2:**
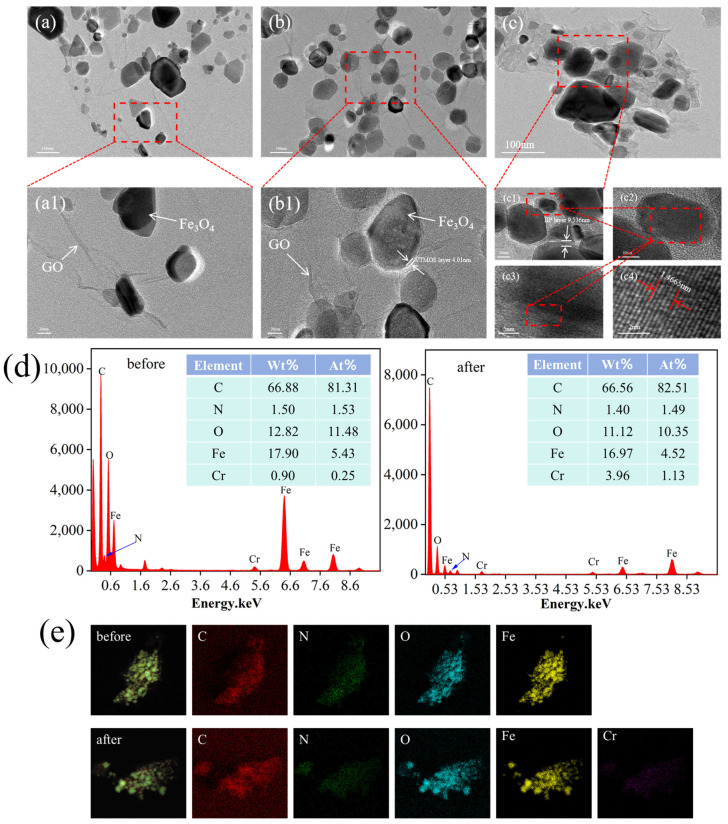
(**a**) TEM images of (**a**) Fe_3_O_4_@GO (**a1** enlarged TEM of **a**), (**b**) Fe_3_O_4_@GO@VTMOS (**b1** enlarged TEM of **b**), (**c**) Fe_3_O_4_@GO@IIP (**c1** of **c**, **c2** of **c1**, **c3** of **c2**, **c4** of **c3** enlarged TEM), (**d**) EDS spectra of Fe_3_O_4_@GO@IIP before and after Cr(VI) adsorption, and (**e**) EDS elemental mapping images of Fe_3_O_4_@GO@IIP before and after Cr(VI) adsorption.

The morphological and structural changes in the samples during adsorbent synthesis were examined using transmission electron microscopy (HRTEM). In Figure 2a, Fe_3_O_4_@GO is depicted, where the black color represents the Fe_3_O_4_ magnetic core exhibiting a smooth surface with an average particle size of approximately 56 nm [24]. The light color corresponds to the GO layer, clearly illustrating the presence of a GO coating surrounding the Fe_3_O_4_ magnetic core, indicating the successful composite formation of Fe_3_O_4_ and GO. Figure 2b displays the HRTEM image of Fe_3_O_4_@GO@VTMOS, showcasing an increase in particle size and a roughened surface post-composite formation. Additionally, a light-colored VTMOS layer approximately 4.01 nm thick is observed on the outer layer of Fe_3_O_4_@GO, confirming the successful composite of VTMOS with Fe_3_O_4_@GO. Upon the incorporation of the imprinted layer, a 9.536 nm imprinted layer is evident on the matrix’s outer surface, as shown in Figure 2c. Furthermore, the lattice gap of the adsorbent was measured to be approximately 0.2933 nm, corresponding to the (220) crystal plane of Fe_3_O_4_ as identified in the XRD analysis [25].

#### 2.1.6. EDS

The EDS elemental spectra analysis revealed the uniform distribution of O and Fe on the surface of the Fe_3_O_4_@GO@IIP material, indicating the successful introduction of the Fe_3_O_4_ magnetic nucleus into the adsorbent. Furthermore, the uniform distribution of the N element indicated the successful functionalization of the adsorbent surface (Figure 2e). Moreover, as depicted in Figure 2d, the Cr element content was 0.25% before adsorption and increased to 1.13% after adsorption. The presence of trace amounts of Cr(VI) before adsorption can be attributed to the template ions encapsulated within the adsorbent’s inner layer during the synthesis process without complete removal. This observation highlights the adsorbent’s effective adsorption capacity [26].

### 2.2. Adsorption Properties

#### 2.2.1. pH Effect on Adsorption

**Figure 3:**
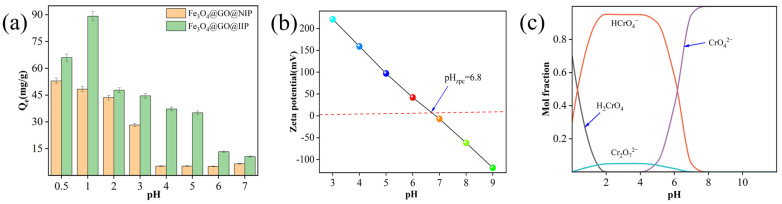
(**a**) The effect of pH on the adsorption capacity of IIP, (**b**) the zeta potential measurements, and the (**c**) species profiles of the Cr(VI)−H_2_O system, 298.15 K.

Considering that Cr(VI) undergoes hydrolytic ionization under acidic conditions, the impact of Fe_3_O_4_@GO@IIP/ Fe_3_O_4_@GO@NIP on the adsorption capacity of 100 mg/L of chromium(VI) at various pH levels at room temperature was examined, as depicted in Figure 3a. The adsorption capacity of chromium(VI) increased gradually with decreasing pH, peaking at 89.18 mg/g at a pH = 1. Zeta potential analysis of Fe_3_O_4_@GO@IIP indicated that the surface of the Fe_3_O_4_@GO@IIP adsorbent became positively charged when the pH was below 6.8 (Figure 3b). As the pH decreased, chromium(VI) existed in various forms such as CrO_4_^2−^, HCrO_4_^−^, Cr_2_O_7_^2−^, and H_2_CrO_4_ (Figure 3c). At a pH = 1, 80% of the chromium(VI) was in the form of HCrO_4_^−^, facilitating electrostatic attraction with the negatively charged HCrO_4_^−^ in the acidic solution [27]. The adsorption capacity decreased after the pH dropped below 1 because at a pH = 0.5, Cr(VI) existed as 50% H_2_CrO_4_ and 50% HCrO_4_^−^, leading to a reduction in the electrostatic effect due to the decrease in HCrO_4_^−^, resulting in a decline in the adsorption capacity. The maximum adsorption capacity of Fe_3_O_4_@GO@NIP was lower than that of Fe_3_O_4_@GO@IIP (52.89 mg/g), attributed to the presence of more adsorption sites on the loose and porous surface of Fe_3_O_4_@GO@IIP. Experimental findings demonstrated that the optimal adsorption efficiency of Fe_3_O_4_@GO@IIP was achieved at a pH = 1.

#### 2.2.2. Adsorption Kinetics

The impact of contact time on the adsorption of Cr(VI) was examined at an initial concentration of 100 mg/L, a pH = 2, and a temperature of 25 °C, as illustrated in Figure S1a. The adsorption of Fe_3_O_4_@GO@IIP reached saturation within 20 min with a saturation capacity of 42.80 mg/g, while Fe_3_O_4_@GO@NIP achieved saturation after 30 min with a saturation capacity of 26.02 mg/g. The lower saturation capacity of Fe_3_O_4_@GO@NIP (26.02 mg/g) can be attributed to the abundance of imprinted cavities in Fe_3_O_4_@GO@IIP, facilitating easier access for the target ions to the adsorption sites. In contrast, Fe_3_O_4_@GO@NIP struggles to efficiently expose its sites due to the dense nature of the polymer coating its surface.

To delve into the kinetic adsorption mechanism between the adsorbent and the target ions comprehensively throughout the adsorption process, the data were nonlinearly simulated using the pseudo-first kinetic model (Equation (1)), the pseudo-second kinetic model (Equation (2)), and the Elovich kinetic model (Equation (3)). Additionally, the data were linearly fitted employing the Weber–Morris model (Equation (4)) [28,29,30,31].

Pseudo-first kinetic model equations:

(1) $$Q_{t}=Q_{e}\left(1-e^{-k_{1}t}\right)$$

Pseudo-second kinetic model equations:

(2) $$Q_{t}=\frac{{Q_{e}}^{2}k_{2}t}{1+Q_{e}k_{2}t}$$

Elovich kinetic model equations:

(3) $$Q_{t}=\frac{1}{k_{3}}\ln\left(ak_{3}t+1\right)$$

Weber–Morris intraparticle diffusion rate equation:

(4) $$Q_{t}=k_{i}t^{0.5}+C$$

where *Q_t_* (mg/g) and *Q_e_* (mg/g) are the adsorption capacity of Cr(VI) at time *t* (min) and equilibrium, respectively, *k*_1_ is the rate constant of the pseudo-first kinetic model, *k*_2_ is the rate constant of the pseudo-second kinetic model at equilibrium, *k*_3_ is the rate constant for the Elovich kinetic model, *a* is the initial adsorption rate, *k_i_* is the rate constant of the Weber–Morris intraparticle diffusion model, and *C* is the boundary layer thickness.

**Figure 4:**
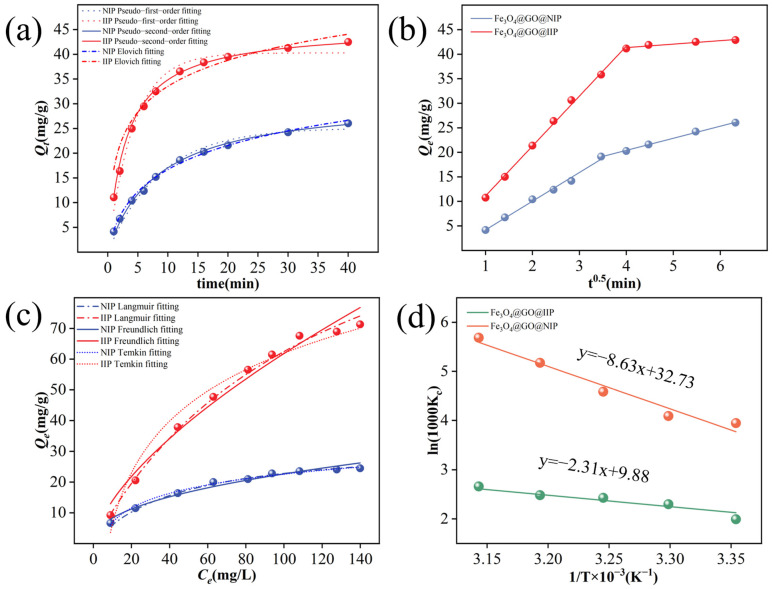
Adsorption kinetic curves (**a**), Weber−Morris curves (**b**), adsorption isotherm curves (**c**) and plot of ln1000K_c_ versus 1/T (**d**) for the IIP and NIP.

The kinetic fitted curve is depicted in Figure 4a, and the relevant parameters are detailed in Table 1. The correlation coefficients of the Fe_3_O_4_@GO@IIP and Fe_3_O_4_@GO@NIP pseudo-second models (R_2_^2^_IIP_ = 0.9963 and R_2_^2^_NIP_ = 0.9912) surpassed those of the pseudo-first models (R_1_^2^_IIP_ = 0.9879 and R_1_^2^_NIP_ = 0.9818) and the Elovich models (R_3_^2^_IIP_ = 0.9554 and R_3_^2^_NIP_ = 0.9820). Furthermore, the computed values of Fe_3_O_4_@GO@IIP’s and Fe_3_O_4_@GO@NIP’s pseudo-second simulations (Q_e,IIP_ = 42.21 mg/g and Q_e,NIP_ = 25.80 mg/g) align more closely with the experimental values (Q_e,IIP_ = 42.80 mg/g and Q_e,NIP_ = 26.02 mg/g). These findings collectively suggest that the adsorption process adheres to pseudo-secondary kinetics and is primarily governed by chemisorption.

The adsorption kinetics of Fe_3_O_4_@GO@IIP and Fe_3_O_4_@GO@NIP were analyzed using the Weber–Morris model (Figure 4b). The absence of the curves intersecting the origin suggests that intraparticle diffusion governs the rate-limiting step and significantly influences the recognition of Cr(VI). Initially, in the diffusion process of Fe_3_O_4_@GO@NIP, Cr(VI) disperses, followed by its attachment to the surface of Fe_3_O_4_@GO@NIP for adsorption. Once the surface adsorption reaches saturation, Cr(VI) further diffuses into the internal channels of Fe_3_O_4_@GO@NIP for additional adsorption. In contrast, the initial diffusion rate of Fe_3_O_4_@GO@IIP is higher than that of Fe_3_O_4_@GO@NIP due to the greater number of available sites on its surface. However, the subsequent diffusion rate of Fe_3_O_4_@GO@IIP is slightly lower than that of Fe_3_O_4_@GO@NIP, likely because most ions are adsorbed during the initial stage, causing a slowdown in diffusion as ion concentration decreases. Ultimately, until adsorption is complete, Cr(VI) fully occupies the binding sites, reaching saturation.

#### 2.2.3. Adsorption Isotherms

To investigate the adsorption behavior during the process, the impacts of varying initial Cr(VI) concentrations on adsorption capacity were examined at a pH = 2, with an adsorption time of 60 min and a temperature of 25 °C. The results are depicted in Figure S1b. It is evident that the adsorption capacity of both Fe_3_O_4_@GO@NIP and Fe_3_O_4_@GO@IIP increased gradually as the initial Cr(VI) concentration rose. Fe_3_O_4_@GO@NIP reached adsorption saturation at 80 mg/L (21.6 mg/g), while Fe_3_O_4_@GO@IIP achieved saturation at 100 mg/L (73.8 mg/g). This disparity can be attributed to the incorporation of a significant number of imprinted sites in Fe_3_O_4_@GO@IIP during its preparation, leading to a substantially higher adsorption capacity compared to Fe_3_O_4_@GO@NIP.

To comprehensively explore the adsorption mechanism in the process, the Langmuir, Freundlich, and Temkin isothermal models were employed to analyze the experimental data, with the corresponding fitting equations presented in Equations (5)–(7) [32,33,34].

Langmuir isothermal equation:

(5) $$Q_{e}=\frac{Q_{m}k_{L}C_{e}}{1+k_{L}C_{e}}$$

Freundlich isothermal equation:

(6) $$Q_{e}=k_{F}{C_{e}}^{\frac{1}{n}}$$

Temkin equation:

(7) $$Q_{e}=\frac{RT}{b}\ln\left(k_{t}C_{e}\right)$$

where *Q_e_* (mg/g) is the adsorption capacity of the Cr(VI) ions at equilibrium, *C_e_* (mg/L) is the equilibrium concentration of the Cr(VI) ions in solution, *Q_m_* (mg/g) is the maximum adsorption capacity of the Cr(VI) ions, and *k_L_* is the constant in the Langmuir model. *k_F_* and n are constants in the Freundlich model, *R* is the gas constant (8.314 J/mol-K), *T* is the temperature, b is Temkin’s constant, and *K_T_* is Temkin’s isothermal constant.

Figure 4c displays the fitting curves of the Langmuir, Freundlich, and Temkin isothermal models, with the corresponding parameters detailed in Table 2. The correlation coefficients (R^2^) for the Langmuir isothermal model were R_1_^2^(IIP) = 0.9994 and R_1_^2^(NIP) = 0.9934, while those for the Freundlich isothermal model were R_2_^2^(IIP) = 0.9772 and R_2_^2^(NIP) = 0.9783, and the correlation coefficients (R^2^) for the Temkin isothermal model were R_3_^2^(IIP) = 0.9738 and R_3_^2^(NIP) = 0.9807. Temkin described Cr(VI) isotherms, suggesting that adsorption may be regulated by a variety of mechanisms. Furthermore, the Langmuir isothermal model predicted adsorption capacities of Q_e,(IIP)_ = 74.06 mg/g and Q_e,(NIP)_ = 24.92 mg/g that were in close agreement with the experimental values of Q_e,(IIP)_ = 73.80 mg/g and Q_e,(NIP)_ = 21.60 mg/g. These results indicate that the adsorption process conforms to surface monolayer adsorption.

#### 2.2.4. Thermodynamic Studies

To explore the impact of temperature on adsorption capacity, the adsorption behavior was studied at a pH = 2, with an adsorption time of 60 min and an initial ion concentration of 100 mg/g. The results are depicted in Figure S1c. The adsorption capacity of Fe_3_O_4_@GO@NIP initially increases and then decreases with rising temperature. On the contrary, the adsorption capacity of Fe_3_O_4_@GO@IIP gradually increased with increasing temperature. This trend may be due to the fact that the adsorption process is heat-absorbing and the increase in temperature can promote the adsorption process. To delve deeper into the thermodynamic aspects of the adsorption process, the free energy ΔG, the enthalpy ΔH, and the entropy ΔS were determined using the Gibbs free energy Equation (8) and the Van ’t Hoff Equation (9) [35].

(8) $$\Delta G=-RTln\left(1000K_{c}\right)$$

(9) $$ln{1000K}_{c}=-\frac{\Delta H}{RT}+\frac{\Delta S}{R}$$

where *R* is the universal gas constant (8.314 J/mol·K), *T* is the absolute temperature (K), and *K_c_* is the (K_c_ = Q_e_/C_e_) equilibrium constant. The value of the Δ*G* was calculated directly from the above relation, and the slope and intercept of *ln1000Kc* versus 1/T were used to obtain the values of the Δ*H* and the Δ*S* as shown in Figure 4d. The calculated values of the thermodynamic parameters are listed in Table 3.

The adsorption of metal ions by the adsorbent was determined to be spontaneous, as indicated by the negative values of the ΔG at various temperatures. In addition, the ΔG decreases with increasing temperature, suggesting that higher temperatures are more favorable for adsorption. The ΔH values for Fe_3_O_4_@GO@IIP and Fe_3_O_4_@GO@NIP were determined to be 0.07180 kJ/mol and 0.01923 kJ/mol, respectively. A positive ΔH value indicates that the adsorption process is heat absorbing and increasing the temperature can promote the adsorption process. In addition, the ΔS values for Fe_3_O_4_@GO@IIP and Fe_3_O_4_@GO@NIP were positive (0.2721 kJ/mol·K and 0.0821 kJ/mol·K), suggesting that there was a transition from an ordered to a disordered state during the adsorption process. Therefore, the adsorption processes of Fe_3_O_4_@GO@IIP and Fe_3_O_4_@GO@NIP are characterized by spontaneous heat absorptions and entropy increases [36].

### 2.3. Selective Adsorption

**Figure 5:**
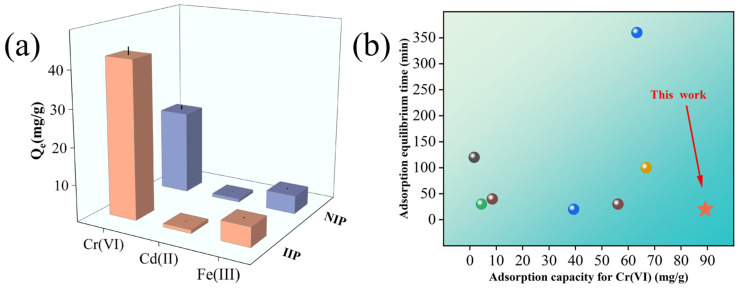
(**a**) The selective adsorption of the IIP and NIP. (**b**) A comparison of other adsorbents.

The selectivity studies aimed to assess the specificity and selectivity of the prepared adsorbent materials. In this context, selective adsorption experiments of Cr(VI) magnetic ion-imprinted polymers were conducted in a ternary solution system, as illustrated in Figure 5a. The Fe_3_O_4_@GO@NIP adsorbent was used as a control in these experiments. The competing ions, Cd(II) and Fe(III), were chosen and adsorbed under the following conditions: a temperature of 25 °C, a pH = 2, an adsorption time of 60 min, and an initial ion concentration of 50 mg/g. Subsequently, the concentrations of each ion after adsorption were determined using ICP-AES. The distribution rate (D), selectivity coefficient (K), and relative selectivity coefficient (K′) were then calculated based on the experimental data. The results of these calculations are presented in Table 4.

The selectivity coefficients k _Cr(VI)/Cd(II)_ and k _Cr(VI)/Fe(III)_ of Fe_3_O_4_@GO@IIP for the ionic competing ions were found to be 290.4796 and 47.2481, respectively. These values indicate the excellent selective adsorption capability of Fe_3_O_4_@GO@IIP in the presence of medium competing ions. Furthermore, the relative selectivity coefficients k′ _Cr(VI)/Cd(II)_ and k′ _Cr(VI)/Fe(III)_ were determined to be 6.4253 and 6.2993, respectively. These results suggest that the adsorption sites of Fe_3_O_4_@GO@IIP exhibit higher affinity and selectivity compared to Fe_3_O_4_@GO@NIP.

To further validate the advantages of the prepared adsorbents, we compared them with previously reported adsorbents in terms of their adsorption capacity and adsorption time for Cr(VI) removal [15,18,37,38,39,40,41]. As shown in Figure 5b, our study achieved a maximum adsorption capacity of 89.8 mg/g and a fast equilibration time of 20 min at 25 °C and pH 1, which exceeded the performance of most previous adsorbents. In addition, the high selectivity of our adsorbent allows it to resist interference from other ions in complex aqueous solutions, while its magnetic properties facilitate simple and rapid subsequent separations by applying an external magnetic field. This is because the use of surface imprinting technology can make the surface of the imprinted material have more adsorption sites, facilitating a reduction in mass transfer and ion transmission during contact with the sites. In conclusion, this material has the potential for the selective separation of Cr(VI).

**Figure 6:**
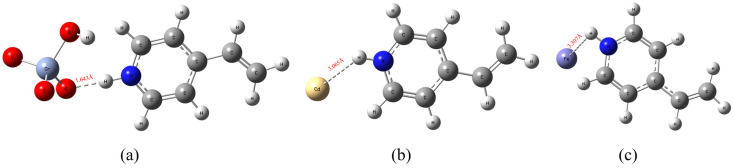
DFT selective bond length calculations for protonated 4VP. (**a**) The HCrO_4_^−^ ion exhibits a protonated 4VP bond, (**b**) Cd(II) displays a protonated 4VP bond, and (**c**) Fe(III) is characterized by a protonated 4VP bond length.

The affinity of Fe_3_O_4_@GO@IIP for the three metal ions was evaluated using density functional theory (DFT), as depicted in Figure 6. The bond length between the protonated 4VP functional monomer and HCrO_4_^−^ was determined to be only 1.643 Å. This short bond length suggests a strong interaction between the functional monomer and the Cr(VI) ion. In contrast, the bond lengths between the protonated 4VP functional monomer and the Cd(II) and Fe(III) ions were measured at 3.065 Å and 3.307 Å, respectively. These longer bond lengths indicate weaker interactions between the functional monomer and the competing ions. The significantly shorter bond length between the 4VP functional monomer and HCrO_4_^−^ compared to those with the Cd(II) and Fe(III) ions highlights the strong affinity of the selected 4VP functional monomers for Cr(VI) in solution. This observation supports the excellent selectivity of Fe_3_O_4_@GO@IIP for Cr(VI) over competing ions.

**Figure 7:**
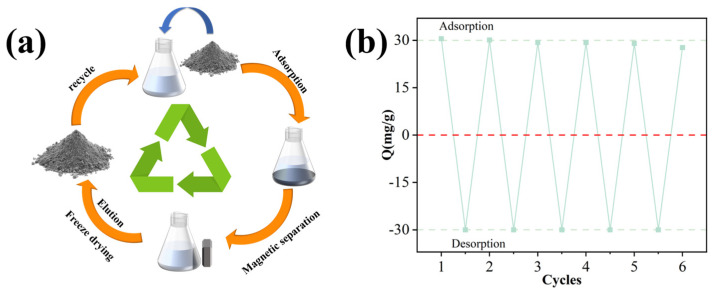
(**a**) The process of recycling the IIP in practical applications. (**b**) The adsorption-desorption cycles of Cr(VI) on the IIP.

A significant advantage of Fe_3_O_4_@GO@IIP over conventional adsorbents is its superior regeneration performance, particularly in the presence of a magnetic field, facilitating the easy separation and recycling of the magnetic adsorbent. This characteristic leads to cost savings and economic benefits. The regeneration process is depicted in Figure 7a, where metal ions are desorbed from the adsorbent, enabling its reuse in subsequent adsorption cycles. As demonstrated in Figure 7b, Fe_3_O_4_@GO@IIP consistently maintained stable adsorption efficiency for Cr(VI) across multiple regeneration cycles.

In conclusion, the pivotal factors for assessing the suitability of an adsorbent for metal ion adsorption are its selectivity and its cyclic regeneration performance. The outstanding performance of Fe_3_O_4_@GO@IIP in these areas substantiates its versatility and significant potential for various applications.

### 2.4. Mechanistic Studies

**Figure 8:**
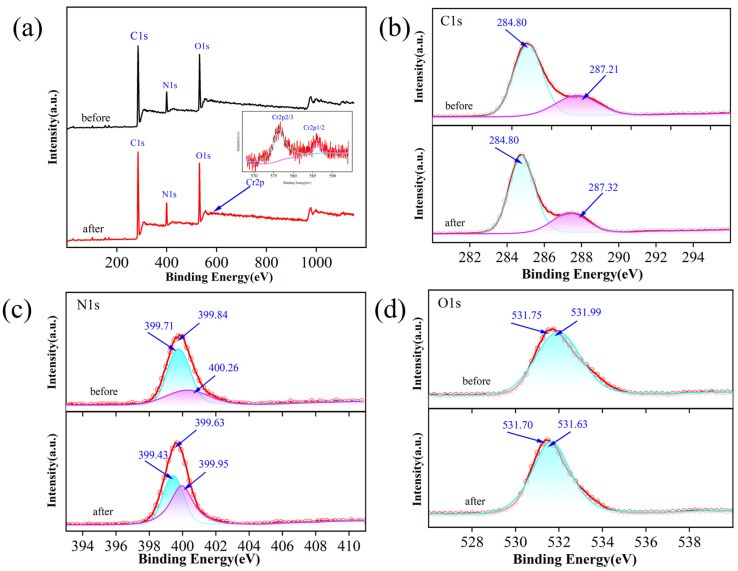
(**a**) XPS spectra of the IIP before and after Cr(II) adsorption, deconvoluted of C1s (**b**), N1s (**c**), and O1s (**d**).

The results of the adsorption experiments, along with the zeta potential plots, indicate the presence of electrostatic interactions in the adsorption process. The adsorption simulation curves further reveal that chemisorption predominates, driven by the protonation of functional monomers on the adsorbent and the ionization hydrolysis of the target metal ions. To delve into the adsorption mechanism from a bonding perspective, X-ray photoelectron spectroscopy (XPS) was utilized to analyze the adsorbent’s specific binding sites before and after adsorption. The distinctive peaks of Cr(VI) were clearly discernible in the wide-scan spectrum both before and after adsorption, with peaks at 577.9 eV and 587.6 eV corresponding to the characteristic absorption peaks of Cr 2p 2/3 and Cr 2p 1/2, respectively, underscoring the adsorbent’s affinity for Cr(VI) (Figure 8a). The subsequent reverse convolution analysis of the C 1s spectrum revealed a slight shift in the C=N peak from 287.21 eV to 287.32 eV post-adsorption, while the C-C peak remained unchanged at 284.80 eV (Figure 8b). The N 1s spectrum exhibited peaks at 399.71 eV and 400.26 eV for N-C and NH^+^ pre-adsorption, which shifted to 399.43 eV and 399.95 eV, respectively, post-adsorption (Figure 8c). In the reverse convolution spectrum of O 1s, the O-Cr peak shifted from 531.99 eV to 531.63 eV after adsorption (Figure 8d). These findings suggest that the adsorption of Cr(VI) on the ion-imprinted polymer (IIP) involves interactions between N atoms on the IIP and O and Cr atoms in the metal ions [42,43,44].

**Figure 9:**
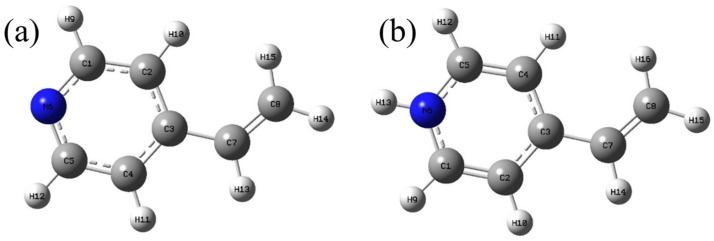
Fukui functional atomic numbers calculated using the implicit solvent (water) model for (**a**) 4VP and (**b**) the ground state of protonated 4VP [pbe1pbe/def2svp].

The adsorption sites on the Fe_3_O_4_@GO@IIP were further elucidated using DFT-calculated Fukui functions to assign nucleophilic and electrophilic capacities to the functional monomer 4VP and its protonated form. These capacities were employed to analyze potential interactions within the structure of the functional monomer, as depicted in Figure 9. Nucleophilicity identifies the sites within the functional monomer that are prone to accepting electrons, particularly regions that exhibit partial or complete positive charges, making them favorable for interacting with negatively charged HCrO_4_^−^. Table S1 reveals that the nucleophilic attack sites in 4VP are primarily associated with the C and N atoms within the pyridine ring, with heightened nucleophilicity observed at f(r)^+^: 6N and 7C, indicating their susceptibility to nucleophilic attack by HCrO_4_^−^. Conversely, calculations for protonated 4VP yielded distinct results, identifying stronger f(r)^+^ nucleophiles at 6N and 13H, leading to altered regions vulnerable to nucleophilic attack compared to the unprotonated 4VP. Notably, regions of the functional monomer where significant changes in f(r)^+^ and f(r)^−^ were not observed are omitted from the analysis [45].

**Figure 10:**
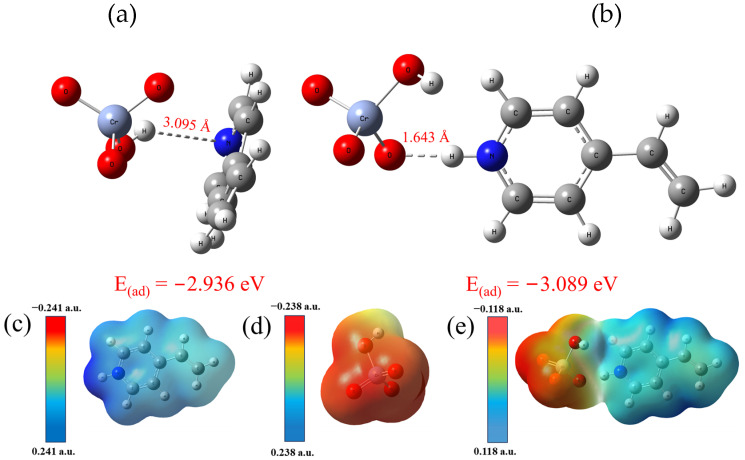
(**a**,**b**) 4VP and protonated 4VP vs. HCrO_4_^−^ binding energies and bond lengths, (**c**−**e**) protonated 4VP, HCrO_4_^−^, and their product’s surface electrostatic formulas.

The binding energies of 4VP and protonated 4VP for HCrO_4_^−^ were calculated using DFT as in Figure 10a,b. The bond length between 4VP and HCrO_4_^−^ is 3.095 Å and the binding energy is −2.936 eV. In contrast, the bond length between protonated 4VP and HCrO_4_^−^ is only 1.643 Å and the binding energy is only −3.089 eV. This also indicates that protonated 4VP has a stronger affinity to bind to HCrO_4_^−^. And, it can be seen from the figure that HCrO_4_^−^ is positioned above of the six-membered ring of 4VP, whereas for protonated 4VP, HCrO_4_^−^ and protonated 4VP lie in the same plane, which is consistent with Fukui’s analysis.

In Figure 10c–e, the 4VP surface exhibits a strong positive charge due to protonation, especially in the vicinity of the N atom, which is due to the fact that the surface protonation carries a positive charge in N under acidic conditions. Cr exists mainly as HCrO_4_^−^ in an aqueous solution. In this state, protonated 4VP exerts an electrostatic attraction on the HCrO_4_^−^ ions in solution. When HCrO_4_^−^ ions enter the adsorption sites formed by protonated N atoms, the two form a stronger bond. The whole complex gradually weakened close to electroneutrality, thus further confirming the existence of electrostatic forces.

**Figure 11:**
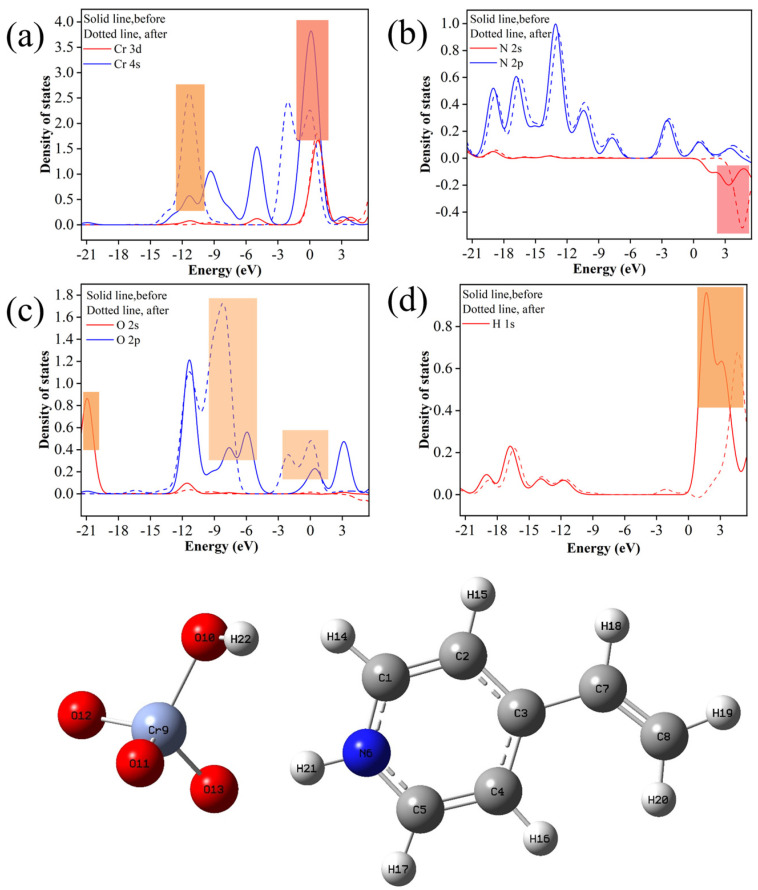
The partial state densities for the following species are presented: (**a**) Cr(VI), (**b**) N6, (**c**) O13, and (**d**) H21.

To investigate the electron transfer during adsorption, the analysis was conducted using the projected density of states (PDOS) (Figure 11). Following adsorption, the Cr 4s orbitals experienced a shift from −13.60 eV to −8.16 eV, with a notable weakening observed at −2.72 eV to 2.72 eV. Conversely, the N 2s orbitals exhibited significant strengthening at 2.72 eV to 0 eV, suggesting electron transfer occurs from HCrO_4_^−^ to the N atoms. Additionally, the O 2s orbital showed a substantial weakening at −21.77 eV to −19.05 eV post-adsorption, while the 2p orbital displayed significant enhancement at −10.88 eV to 2.72 eV. Furthermore, a shift in the 1s orbital of H from −2.72 eV to 5.44 eV was observed after adsorption, indicating the formation of bonds between O and H atoms, thereby enhancing the stability of the adsorption product. The electron transfer observed during adsorption implies that the process proceeds via chemisorption [46].

By conducting a precise wave function analysis to predict the charge transfer quantities of protonated 4VP and HCrO_4_^−^ (Table S2), the results showed that HCrO_4_^−^ lost 0.2008 eV of total net electrons during adsorption. Furthermore, the surface HCrO_4_^−^ transfers its electrons to the protonated 4VP, consistent with the findings of the localized density of states analysis (PDOS). This observation reinforces the assertion that the adsorption process involves chemisorption and is accompanied by charge transfer.

### 2.5. Practical Applications

To assess the potential of the synthesized Fe_3_O_4_@GO@IIP materials for practical utilization in industrial tailings, 20 mg of the adsorbent was introduced into 20 mL of nitrified industrial wastewater and agitated at room temperature for 60 min to facilitate the removal of chromium from the nitrified industrial effluent. The subsequent analysis showed that the concentration of Cr(VI) in the treated industrial tailwater was reduced from 5.989 mg/L to only 0.0829 mg/L, thereby meeting the WHO discharge standard of less than 0.1 ppm post-treatment. The Fe_3_O_4_@GO@IIP showed an excellent selective adsorption capacity even in the real industrial tailwater, which highlighted its great potential for practical application.

## 3. Materials and Methods

### 3.1. Reagents

The reagents used are detailed in the Supplementary Materials (S1).

### 3.2. Instruments and Equipment

The models and manufacturers of the instruments used are detailed in the Supplementary Materials (S2).

### 3.3. Preparation of Imprinted Materials

**Figure 12:**
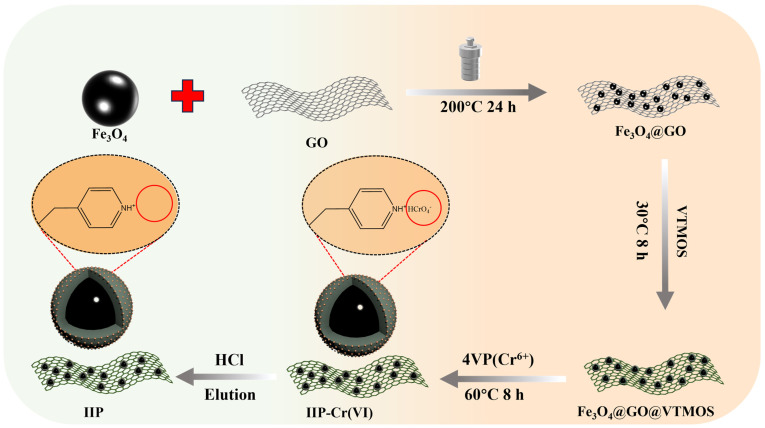
The detailed synthetic route of Fe_3_O_4_@GO@IIP-Cr(VI).

The detailed synthetic route of Fe_3_O_4_@GO@IIP-Cr(VI) is shown below (Figure 12).

#### 3.3.1. Preparation of Magnetic Fe_3_O_4_@GO Nanocomposite Matrices

The Fe_3_O_4_@GO synthesis procedure is detailed in the Supplementary Materials (S3).

#### 3.3.2. Preparation of Graphene-Based Magnetic Cr(VI) Nano-Imprinted Polymers

An amount of 0.2 g of Fe_3_O_4_@GO was dispersed ultrasonically in 150 mL of anhydrous ethanol. Subsequently, 1 mL of vinyltrimethoxysilane (VTMOS) and 10 mL of ammonia were added dropwise to the dispersion under mechanical stirring. The reaction proceeded at 30 °C for 8 h. The resulting products were separated using a magnet, washed thrice with methanol, and then dried under a vacuum at 40 °C in a vacuum drying oven for 24 h to yield the modified Fe_3_O_4_@GO@VTMOS.

In a three-necked flask, 1 mL of K_2_Cr_2_O_7_ (5 g/L) and 2 mL of tetravinylpyridine (4VP) were dissolved in 30 mL of methanol and magnetically stirred at room temperature for 30 min. Subsequently, 0.2 g of Fe_3_O_4_@GO@VTMOS was added and stirred for 12 h. Another solution of K_2_Cr_2_O_7_ (5 g/L) and 4VP in 30 mL of methanol was prepared. Then, 1.6 mL of 2-hydroxyethyl methacrylate (HEMA), 0.7 mL of ethylene glycol bis(methacrylate) (EGDMA), and 0.1 g of azobisisobutyronitrile (AIBN) were added to the mixture, and the reaction was conducted for 8 h in a water bath heated at 60 °C. After the reaction, the products were extracted using a magnet, and the polymerized product was washed with deionized water to eliminate unreacted cross-linkers and functional monomers. Subsequently, it was washed with 2 mol/L of HCl to completely remove template Cr(VI) ions. The polymerized product was further washed with deionized water to remove any remaining unreacted cross-linkers and functional monomers, followed by washing with 2 mol/L of HCl to ensure the complete removal of template Cr(VI) ions. Upon the completion of leaching, Fe_3_O_4_@GO@IIP(IIP) was obtained via neutral washing with deionized water and subsequent vacuum drying.

Fe_3_O_4_@GO@NIP(NIP) was prepared using the same method, with the exception that no template ions were included as a control during polymerization.

### 3.4. Adsorption Experiments

Fe_3_O_4_@GO@IIP was utilized as the adsorbent material, and 20 mg of the adsorbent was introduced into 20 mL of metal ion solutions with varying concentrations for conducting batch adsorption experiments. The mixtures were agitated at 25 ± 1 °C for different durations, and the metal ion concentrations were analyzed using ICP-AES. Each adsorption experiment was conducted in triplicate, and the average value was computed subsequently to reduce experimental discrepancies. The adsorption capacity was determined based on Equation (10) [47].

(10) $$Q_{e}=\frac{C_{0}-C_{e}}{1000W}\times V$$

In the context of the adsorption process, *Q_e_* (mg/g) represents the adsorption capacity, while *C*_0_ (mg/L) and *C_e_* (mg/L) stand for the initial and final concentrations of the metal ions post-adsorption, respectively. Additionally, *V* (mL) denotes the volume of the solution, and *W* (g) signifies the mass of the adsorbent utilized.

The study investigated the influence of pH, time, temperature, and initial concentration on the adsorption capacity of Cr(VI). Initially, 20 mL of a 100 mg/L Cr(VI) solution was added to a 150 mL stoppered conical flask, with pH levels adjusted to 0.5, 1, 2, 3, 4, and 5. Subsequently, 20 mg of adsorbent samples were introduced, and the adsorption process occurred for 1 h in a water-bath shaker at 25 °C. Analysis using ICP-AES determined the concentration of the adsorbed Cr(VI). The impact of time on adsorption capacity was also explored by collecting samples at 1, 2, 4, 6, 8, 12, 16, 20, 30, and 40 min intervals during adsorption at 25 °C. The post-adsorption concentration of Cr(VI) was measured using ICP-AES. To assess the effect of temperature, the adsorption process was conducted at temperatures of 25, 30, 35, 40, and 45 °C. Samples were collected and analyzed for the concentration of adsorbed Cr(VI) using ICP-AES. Finally, the impact of the initial concentration was studied by adding Cr(VI) solutions with concentrations ranging from 10 to 140 mg/L to 150 mL stoppered conical flasks. After agitation for 1 h at 25 °C in a water-bath shaker, the concentration of adsorbent post-adsorption was determined using ICP-AES, and the Cr(VI) concentration post-adsorption was also analyzed.

The selective adsorption experiment involved using a 20 mL solution of a ternary system containing Cr(VI), Cd(II), and Fe(III) at a concentration of 50 mg/L. Subsequently, 20 mg of the adsorbent sample was added, and the adsorption process was carried out for 1 h in a water-bath shaker at 25 °C. The concentrations of the adsorbed ions were analyzed using ICP-AES. The distribution ratio (D), selectivity coefficient (K), and relative selectivity coefficient (K′) were determined by applying Equations (11)–(13), respectively [48].

(11) $$D=\frac{C_{0}-C_{e}}{C_{e}}\times \frac{V}{W}$$

(12) $$k=\frac{D_{Cr}}{D_{M}}$$

(13) $$k^{’}=\frac{k_{IIP}}{k_{NIP}}$$

where *C_0_* (mg/L) and *C_e_* (mg/L) represent the initial and equilibrium concentrations of metal ions, respectively. *V* (mL) denotes the volume of the metal ion solution, *W* (g) stands for the mass of the adsorbent, and *M* signifies the other competing metal ions.

### 3.5. DFT Calculations

DFT calculations were conducted using Gaussian16 to determine the adsorption energies [49]. The aqueous system was optimized with the PBE1PBE/def2svp basis set and the D3 (BJ) dispersive energy correction. Single-point energy calculations were executed post geometry optimization [50]. The regions of 4VP and protonated 4VP susceptible to nucleophilic and electrophilic attack were identified using the Fukui function, denoted as f(r)^+^ and f(r)^−^, respectively. The Multiwfn multifunctional wavefunction analysis program was employed for wavefunction analyses and correlation image plotting. The adsorption energy (E_ad_) between the 4VP and the protonated 4VP with metal ions was determined using Equation (14).

(14) $$E_{\left(ad\right)}=E_{\left(total\right)}-E_{\left(4VP\right)}-E_{\left(adsorbates\right)}$$

where *E*_(*total*)_, *E*_(*4VP*)_, and *E*_(*adsorbates*)_ represent the total energy of the adsorption complex, 4VP, and the adsorbate, respectively.

## 4. Conclusions

In this study, we prepared a magnetic nano-imprinted adsorbent (Fe_3_O_4_@GO@IIP). The successful preparation of Fe_3_O_4_@GO@IIP was confirmed by FT-IR, XRD, EDS, and other characterization techniques. Due to the strong covalent bonding between the materials, it shows excellent structural stability in harsh environments. The excellent adsorption performance of Fe_3_O_4_@GO@IIP on Cr(VI) in a strongly acidic solution (pH = 1) was confirmed by batch adsorption experiments and model simulations, with the maximum adsorption capacity reaching 89.18 mg/g. Correspondingly, the excellent adsorption performance (adsorption capacity, selectivity, and kinetics) of Fe_3_O_4_@GO@IIP on Cr(VI) was attributed to the protonated, strong electrostatic and coordination effects of pyridine nitrogen on Cr(VI). The nucleophilic reaction regions of 4VP and protonated 4VP were assigned and their possible reaction sites were predicted by XPS and DFT calculations. The surface electrostatic formulae before and after the reaction of Fe_3_O_4_@GO@IIP with HCrO_4_^−^ were analyzed to verify the existence of electrostatic interactions in the adsorption process. The charge transfer that occurs between Fe_3_O_4_@GO@IIP and HCrO_4_^−^ during the adsorption process was analyzed using local state density and CDA to verify the presence of chemisorption during the adsorption process. In summary, in this study, Fe_3_O_4_@GO@IIP magnetic imprinted materials were prepared using magnetic separation and surface imprinting techniques, the successful preparation of Fe_3_O_4_@GO@IIP was verified using various characterization techniques, and its adsorption conditions were optimized using batch adsorption experiments. The mechanism of the electrostatic synergistic chemisorption of Cr(VI) by Fe_3_O_4_@GO@IIP in a strong acidic system (pH = 1) was revealed by the model simulation and DFT calculations, which provides a new idea for the further realization of the harmless treatment of trace heavy metal solutions as well as the practical treatment of strongly acidic industrial wastewater.

## Figures and Tables

**Table 1:** The adsorption kinetic parameters of the IIP and NIP.

Adsorbents	Q_e_,exp	Pseudo−First−Order			Pseudo−Second−Order			Elovich			
		Q_e_	k_1_	R_1_^2^	Q_e_	k_2_	R_2_^2^	Q_e_	k_3_	R_3_^2^	a
IIP	42.80	40.27	0.2337	0.9879	42.21	0.0072	0.9963	44.05	0.1306	0.9554	4.832
NIP	26.02	24.87	0.1154	0.9818	25.80	0.0039	0.9912	26.50	0.1303	0.9820	5.997

**Table 2:** The isotherm model parameters used for the IIP and NIP.

Adsorbents	Q_e_,exp	Langmuir			Freundlich				Temkin		
		Q_e_	k_L_	R_1_^2^	Q_e_	k_F_	n	R_2_^2^	Q_e_	k_t_	R_3_^2^
IIP	73.80	74.06	0.0082	0.9994	76.54	3.1417	1.5457	0.9772	69.87	0.1299	0.9737
NIP	21.60	24.92	0.0227	0.9934	26.20	3.0431	2.2934	0.9783	24.50	0.2734	0.9807

**Table 3:** The adsorption thermodynamic parameters for the IIP and NIP.

Adsorbent	Temperature (K)	ΔG (kJ/mol)	ΔH (kJ/mol)	ΔS (kJ/mol·K)
IIP	298	−10.4299	0.07180	0.2721
	303	−10.6399		
	308	−11.7364		
	313	−13.0324		
	318	−14.0872		
NIP	298	−5.2681	0.01923	0.0821
	303	−5.8866		
	308	−6.1461		
	313	−6.3207		
	318	−6.7006		

**Table 4:** Selectivity Parameters of the IIP and NIP.

Metal Ion	IIP			NIP			k′
	Q_e_ (mg/g)	D (mL/g)	k	Q_e_ (mg/g)	D (mL/g)	k	
Cr(VI)	42.5300	5693.4		22.570	822.8		
Cd(II)	0.9610	19.6	290.4796	0.8970	18.2	45.2088	6.4253
Fe(III)	5.3787	120.5	47.2481	4.9435	109.7	7.5005	6.2993

## Data Availability

Data are available within the article and Supplementary Materials.

## Supplementary Materials

The following supporting information can be downloaded at: https://www.mdpi.com/article/10.3390/molecules29091952/s1, S1 reagents, S2 Instruments and equipment, S3 Preparation of magnetic Fe_3_O_4_@GO nanocomposite matrices, Figure S1. (a) Effect of time on adsorption, (b) Effect of initial Cr(VI) concentration on adsorption, (c) Effect of temperature on adsorption; Table S1. Correlated calculated values of the Fukui function for sites susceptible to nucleophilic [f(r)^+^] and electrophilic [f(r)^−^] attack by 4VP and protonated 4VP. Atoms are labeled as in Figure 9a,b; Table S2. The extended charge decomposition analysis (CDA) results for protonated 4VP-Cr(VI).
