# Supplementary material for: Magnetic Ion-Imprinted Materials for Selective Adsorption of Cr(VI): Adsorption Behavior and Mechanism Study
Source: Molecules. 2024 Apr 24;29(9):1952. doi: 10.3390/molecules29091952 (PMC11085326; doi:10.3390/molecules29091952)
Supplement: Supplementary file 1 [file molecules-29-01952-s001.zip › molecules-2966616-supplementary.pdf]

Supplementary Material

for

Magnetic Ion-Imprinted Materials for Selective Adsorption of Cr (VI): Adsorption Behavior and Mechanism Study

Shunfei Li, Siqing Ye, Weiye Zhang, Hongxing He *, Yi Zhang, Mingyang Xiong, Yuhan Chen, Mingqiu Wang and Zhifeng Nie *

Yunnan Key Laboratory of Metal-Organic Molecular Materials and Device, School of Chemistry and Chemical Engineering, Kunming University, Kunming 650214, China

* Correspondence: hxhe0212@kmu.edu.cn (H.H.); niezf123@163.com (Z.N.)

S1 Reagents

$FeCl_3·6H_2O$ and $FeSO_4·7H_2O$ were purchased from Windship Chemical Reagent Technology Co. Ltd (Tianjin, China). Cd $(NO_3)_2·4H_2O$ was sourced from Beijing Chemical Factory (Beijing, China). K_2CrO_7 was purchased from Chengdu Colony Chemicals Co. Biochemical Technology Co. (Shanghai, China). Graphene oxide (GO) was obtained from Macklin Biochemical Technology Co. (Shanghai, China). All chemicals utilized in the experiments met analytical grade standards or higher. Deionized water was employed in the preparation of all aqueous solutions

S2 Instruments and Equipment

The pH was measured with a pH meter (PHS-3C, NESA, Shanghai). Infrared measurements were conducted using a Fourier Transform Infrared (FTIR) spectrometer (Cary 6400, Agilent). Sample morphology and microstructure were examined with a high-resolution transmission electron microscope (HRTEM; JEOL JEM-2100, Hitachi). Thermal stability analysis was performed through thermogravimetric analysis (TG; Hitachi). X-ray diffraction (XRD) patterns were obtained using an X-ray diffractometer (D2 PHASER, Bruker). X-ray photoelectron spectroscopy (XPS) analyses were carried out with a Thermo Escalab 250 Xi spectrometer. Metal ion concentrations were determined by inductively coupled plasma atomic emission spectrometry (ICP-AES; Icap-6300, Thermo Scientific). Magnetic field strength was measured using a vibrating sample magnetometer (VSM; MPMSXL, Quantum Design).

S3 Preparation of Magnetic Fe_3O_4@GO Nanocomposite Matrices

Initially, 12 g of $FeCl_3·6H_2O$ and 6.116 g of $FeSO_4·7H_2O$ were dissolved ultrasonically in 100 mL of deionized water, and the solution was then transferred to a 250 mL three-necked flask. The experimental setup was arranged, and the mixture was stirred under a nitrogen atmosphere for 30 minutes at room temperature. Subsequently, the pH was adjusted to 11 by adding ammonia, and the reaction proceeded under nitrogen and heating at 60°C for 1 hour. Following this, the reaction was allowed to proceed at room temperature for an additional 2 hours. The resulting product was washed with deionized water until neutral, dried, and subsequently ground to yield Fe_3O_4 nanoparticles.

Subsequently, 0.2 g of Fe_3O_4 was dissolved ultrasonically in 30 mL of anhydrous ethanol and dispersed through ultrasonication for 1 hour. Following this, two drops of 2-acryloyl-2-methylpropionic acid (APTES) were introduced to the solution and thoroughly stirred for 2 hours. Subsequently, 10 mL of a 10 g/L aqueous graphene oxide (GO) solution was slowly added dropwise to the mixed reaction solution. The resulting mixture was then transferred to an autoclave and heated to 200 ± 1 °C for 24 hours. The reaction was conducted within the autoclave. Upon cooling, the black solid product was retrieved using a magnet, washed multiple times with ethanol and subsequently with deionized water, and finally dried under vacuum at 60 °C for 12 hours to yield Fe_3O_4@GO.

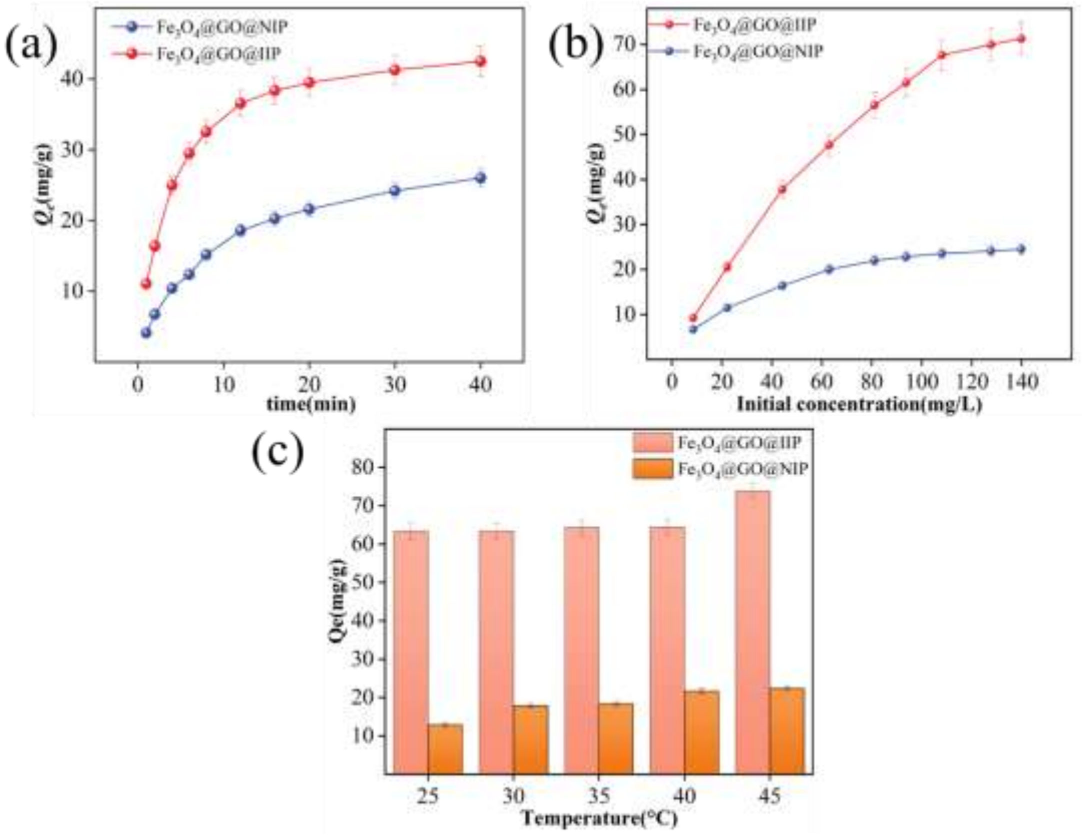

Figure. S1 (a) Effect of time on adsorption, (b) Effect of initial Cr (VI) concentration on adsorption, (c) Effect of temperature on adsorption.

Table S1. Correlated calculated values of the Fukui function for sites susceptible to nucleophilic [f(r)$^+$] and electrophilic [f(r)$^-$] attack by 4 VP and protonated 4VP. Atoms are labeled as in Figure 9 (a) and (b).

Number	Atom	f(r)$^+$	f(r)$^-$	Number	Atom	f(r)$^+$	f(r)$^-$
6	N	0.125	0.117	6	N	0.092	0.065
7	C	0.069	0.179	7	C	0.009	0.271
9	H	0.025	0.022	9	H	0.028	0.017
12	H	0.027	0.022	12	H	0.031	0.017
				13	H	0.035	0.018

Table S2. extended charge decomposition analysis (CDA) results for protonated 4VP-Cr (VI).

Orb.	d	b	b-d	r
1	0	-0.000001	0.000001	0
2	0	0.000013	-0.000013	0
3	0	-0.000001	0.000001	0
4	0	-0.000002	0.000002	0
5	0	0.000001	-0.000001	0
6	0	0.000022	-0.000023	-0.000001
7	0	-0.000001	0	0
8	0	-0.000003	0.000003	0
9	-0.000001	0.000272	-0.000273	0.000016
10	-0.000029	-0.000009	-0.00002	-0.000015
11	-0.000001	0	-0.000001	-0.000001
12	-0.000001	-0.000001	-0.000001	-0.000003
13	0	0	0	0
14	0	0	0	0
15	0.000001	0	0.000001	0
16	0	0	0	0
17	0	0	0	0
18	-0.000001	0.000348	-0.000349	0.000008
19	-0.000001	0.000199	-0.0002	0.000008
20	0	0.00004	-0.00004	0.000004
21	-0.000005	-0.000028	0.000024	-0.000006
22	-0.000279	0.000069	-0.000348	0.002298
23	-0.000008	0.002677	-0.002685	0.00241
24	0.000157	-0.000172	0.00033	0.003196
25	0.000037	0.007663	-0.007626	0.019679
26	0.000134	0.000038	0.000096	-0.000149
27	-0.000086	0.00426	-0.004346	0.012144
28	-0.000005	0.000075	-0.00008	0.000161
29	0.0002	0.001847	-0.001646	-0.008113
30	0.00011	0.000218	-0.000109	-0.001322
31	0.000122	0.000224	-0.000102	-0.00197
32	0.001257	0.000388	0.000869	0.000407
33	0.000477	0.000593	-0.000117	0.00023
34	-0.000024	0.00002	-0.000044	0.000482
35	-0.000276	0.000004	-0.00028	0.003199
36	0.000198	-0.000046	0.000244	0.003124
37	0.000361	-0.000042	0.000404	0.003887
38	0.000246	0.000005	0.000241	0.001226
39	-0.00013	-0.00005	-0.000081	0.002849

40	-0.000523	-0.000138	-0.000385	0.010485
41	0.000015	0.0006	-0.000584	0.003686
42	-0.000015	0.000064	-0.000079	-0.003461
43	-0.000013	0.000031	-0.000044	0.001312
44	0.000243	-0.000022	0.000265	0.000532
45	0.000525	0.015924	-0.015399	-0.019176
46	-0.000027	0.000581	-0.000607	-0.000943
47	0.000099	0.004478	-0.00438	-0.001192
48	0.001186	0.034069	-0.032883	-0.038244
49	0.00058	0.000745	-0.000165	-0.002135
50	-0.000111	0.000423	-0.000534	-0.001708
51	0.000159	-0.000038	0.000197	0.000814
52	-0.000074	0.002999	-0.003073	-0.002193
53	0.000746	0.032337	-0.031591	-0.028732
54	0.000342	0.018757	-0.018415	-0.022138
55	-0.000014	0.003441	-0.003454	-0.003858
56	-0.000378	0.007923	-0.008301	-0.013638
57	0.000026	0.002528	-0.002502	-0.002121
58	0	0	0	0
59	0	0	0	0
60	0	0	0	0
61	0	0	0	0

d = The number of electrons donated from fragment 1 (HCrO4-) to fragment 2 (Protonation 4VP)

b = The number of electrons back donated from fragment 2 (Protonation 4VP) to fragment 1 (HCrO4-)

r = The number of electrons involved in repulsive polarization

Extended Charge decomposition analysis (ECDA)

Contribution to all occupied complex orbital:

Occupied, virtual orbitals of fragment 1: 2798.3347% 11.7046%

Occupied, virtual orbitals of fragment 2: 2888.9294% 1.0312%

Contribution to all virtual complex orbital:

Occupied, virtual orbitals of 4-VP: 1.6653% 12388.2954%

Occupied, virtual orbitals of HCrO4: 11.0706% 6298.9688%

PL(1) + CT(1-> 2) = 0.0333 PL(1) + CT(2-> 1) = 0.2341

PL(2) + CT(1-> 2) = 0.0206 PL(2) + CT(2-> 1) = 0.2214

The net electrons obtained by fragment 1 = CT(1-> 2) - CT(2-> 1) = -0.2008
